# MR Neurography: Advances

**DOI:** 10.1155/2013/809568

**Published:** 2013-03-26

**Authors:** Avneesh Chhabra, Lianxin Zhao, John A. Carrino, Eo Trueblood, Saso Koceski, Filip Shteriev, Lionel Lenkinski, Christopher D. J. Sinclair, Gustav Andreisek

**Affiliations:** ^1^Musculoskeletal Radiology Section, Russell H Morgan Department of Radiology & Radiological Science, Johns Hopkins University Hospital, Baltimore, MD 21287, USA; ^2^Department of MR, Shandong Medical Imaging Research Institute, Shandong University, China; ^3^Department of Intelligent Systems and Robotics, Faculty of Computer Science, University Goce Delcev Stip, 2000 Stip, Macedonia; ^4^SyNRG Software & IT Solutions, Skopje, Macedonia; ^5^3D Imaging Partners, Toronto, ON, Canada; ^6^MRC Centre for Neuromuscular Diseases, UCL Institute of Neurology, University College London, Queen Square, London WC1N 3BG, UK; ^7^Department of Radiology, University Hospital Zurich, Zurich, Switzerland

## Abstract

High resolution and high field magnetic resonance neurography (MR neurography, MRN) is shown to have excellent anatomic capability. There have been considerable advances in the technology in the last few years leading to various feasibility studies using different structural and functional imaging approaches in both clinical and research settings. This paper is intended to be a useful seminar for readers who want to gain knowledge of the advancements in the MRN pulse sequences currently used in clinical practice as well as learn about the other techniques on the horizon aimed at better depiction of nerve anatomy, pathology, and potential noninvasive evaluation of nerve degeneration or regeneration.

## 1. Introduction

High resolution and high field (3T) magnetic resonance neurography (MR neurography, MRN) has been shown to have excellent anatomic capability. This is in part due to the rapid improvements in coil technology and software in the last few years [1–3]. With improved detection of nerve anatomy and pathology, the value of quantitative functional MR methods as potential biomarkers in neuromuscular disease is also being increasingly recognized [4]. While high resolution 2D (dimensional) and 3D demonstration of peripheral nerve anatomy and pathology is the current state of the art, there have been considerable advances in various aspects of the field leading to successful feasibility studies employing various structural and functional imaging techniques in both clinical and research settings [1, 5–7]. This paper is intended to be a useful seminar for readers who want to gain knowledge of the advancements in the MRN pulse sequences currently used in clinical practice as well as learn about the other techniques on the horizon aimed at better depiction of nerve anatomy, pathology, and potential noninvasive evaluation of nerve degeneration or regeneration.

## 2. Clinical Need for MR Neurography

It is estimated that about 5% of population has some form of neuropathy, and there is up to 5% incidence of peripheral nerve injuries in admissions to a level I trauma center [8, 9], with Sunderland grade I–IV injuries (nerve in continuity) far more common than the grade V injury (nerve discontinuity). Clinical evaluation and electrodiagnostic studies provide incomplete information about the anatomy and degree of injury. In particular, these studies are not able to differentiate among grade III–V injuries, which is important from a surgical point of view and patient prognosis [10, 11]. Many of these patients undergo conservative management for 1-2 years depending upon the clinical scenario. One has to often weigh the benefit versus risk of expectant waiting during this period being cognizant of the fact that in the unfortunate event of poor regenerative response and no recovery, the regional muscles will atrophy and tendon transfers as well as consequent disability will be the only option left for the patient with a poor outcome [12]. High field (3 Tesla, T) imaging employing high resolution MRN techniques is an important noninvasive modality that can both detect as well as help to grade nerve injuries [2, 13].

In the clinical arena, there is also an immediate need for reliable quantitative measures that can evaluate peripheral nerve regeneration/further degeneration in patients with traumatic nerve injuries. Proximodistal axonal regeneration is the leading mechanism of recovery from nerve injuries. However, it occurs at a slow pace (~1 mm/day), and the only available clinical tool of “advancing Tinel's sign” is often unreliable, subjective, and frequently inaccurate. The electrodiagnostic studies are also not sufficient due to their complete dependence on target innervation and lack of interobserver reliability [12]. Therefore, it is crucial for development of a technique that can detect the nerve continuity or neuroma and can be used serially in a noninvasive manner to detect the initiation as well as follow the regeneration in injured axons proximally and across the injury site. Another immediate clinical and preclinical need for noninvasive outcome measurement of nerve regeneration is in the area of therapeutics aiming to enhance nerve growth in injuries and mono-/polyneuropathy conditions. Availability of reliable noninvasive techniques can facilitate clinical trials using nerve regeneration enhancers (neurotrophic agents), improvements in the development of nerve conduits with incorporated molecular biology agents, such as stem cells, proteins, and nanotechnology [14–16].

## 3. Three-Dimensional Anatomic Nonselective MR Neurography

Three-dimensional (3D) anatomic MR imaging is not new and has been around for more than 2 decades. However, the latest 3D imaging techniques encompass high resolution (base resolution > 256 and in plane resolution ~1 mm) with superior T2 type contrast also available in some sequences. Additional advanced characteristics include absence of pulsation artifacts and isotropic acquisition with multiplanar capabilities leading to no or only little loss of tissue definition on secondary reformatted images. The addition of recently developed 3D imaging techniques to standard 2D MR imaging protocols helps to overcome difficulties such as imprecise lesion localization, tissue characterization, partial volume artifacts, and overall image interpretation [1, 4, 13, 17].

**Figure 1:**
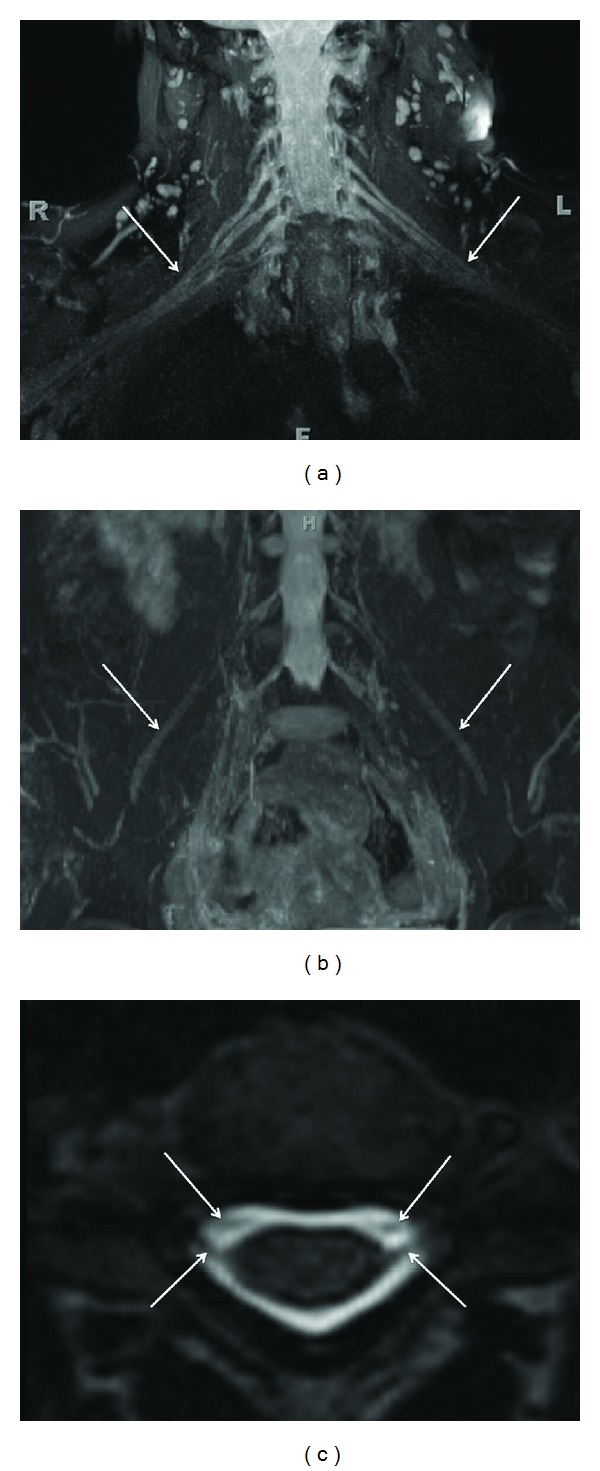
(a) 3D anatomic nonselective MRN-MIP reconstruction from a coronal 3D STIR SPACE sequence shows normal symmetric signal intensity and size of bilateral brachial plexuses (arrows). (b) 3D anatomic nonselective MRN-MIP reconstruction from a coronal 3D STIR SPACE sequence shows normal symmetric signal intensity and size of bilateral LS plexuses and femoral nerves (arrows). (c) 3D anatomic nonselective MRN-Axial reconstruction from 3D T2 SPACE sequence shows the dorsal and ventral roots (preganglionic segments) on both sides (arrows).

**Figure 2:**
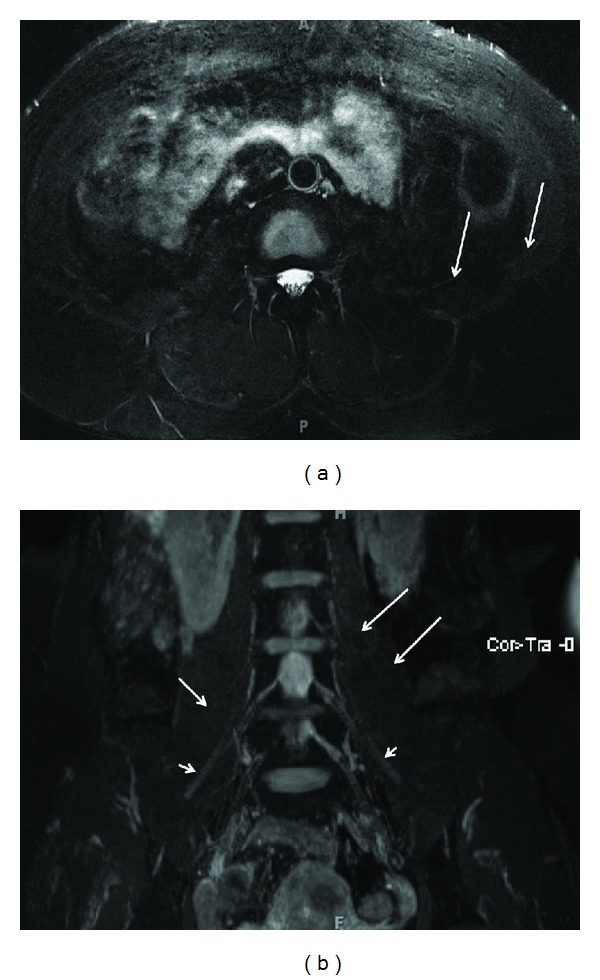
(a) 2D versus 3D anatomic nonselective MRN-Axial T2 SPAIR image through the lower abdomen shows the abnormally hyperintense left ilioinguinal nerve (arrows) in a suspected case of left ilioinguinal neuropathy. (b) 2D versus 3D anatomic nonselective MRN-MIP reconstruction from a coronal 3D STIR SPACE sequence barely shows the left ilioinguinal nerve (large arrows). Notice normal right ilioinguinal nerve (medium arrow) and normal bilateral femoral nerves (small arrows).

**Figure 3:**
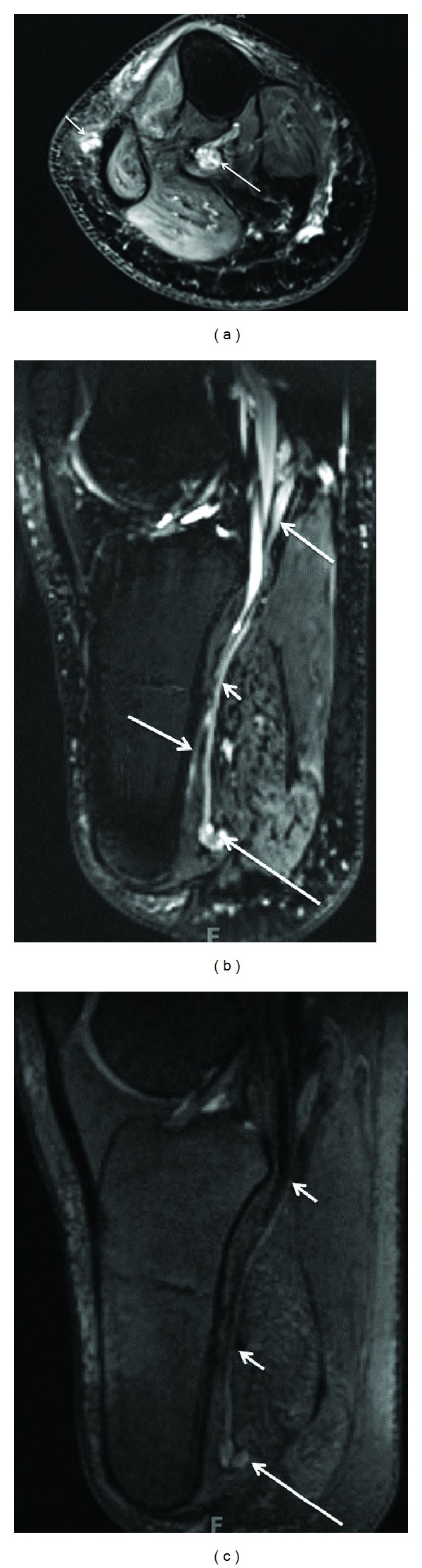
(a) Limitation of 3D anatomic nonselective MRN-Axial T2 SPAIR image through the upper calf shows amputation neuromas of tibial nerve (large arrow) and common peroneal nerve (small arrow). (b) Limitation of 3D anatomic nonselective MRN-Sagittal reconstruction from a 3D SPAIR SPACE sequence in the same case shows the abnormal tibial nerve in its long axis (smaller arrow) with an end-bulb neuroma (large arrow). However, there is suboptimal depiction due to hyperintense vascular signal contamination (medium arrows). (c) 3D anatomic nerve selective MRN-Sagittal reconstruction from a 3D DW PSIF sequence in the same case selectively shows the abnormal tibial nerve in its long axis (small arrows) with an end-bulb neuroma (large arrow).

These new 3D anatomic MR sequences are now possible due to increased signal-to-noise ratio (SNR) of the high field MR scanners, improved coil design and prudent use of parallel imaging techniques. Parallel imaging helps achieve faster acquisition, increased contrast resolution and/or number of slices, better background fat suppression, and a reduction in the echo time/echo spacing, ultimately resulting in less image distortion [1, 17]. On current 3T MR scanners, 3D imaging can be quickly obtained within 5-6 minutes depending upon the field of view and isotropic resolution set in the range of 0.9–1.5 mm. With curved planar reformations (CPRs) and maximum intensity projections (MIPs), there is further reduction of noise, and the peripheral nerves can be beautifully displayed along their long axis to demonstrate the nerve anatomy and pathology [18]. These sequences are termed differently by various vendors as SPACE (sampling perfection with application optimized contrasts using varying flip angle evolutions, Siemens), Cube (General Electric Healthcare, Waukesha, WI), and VISTA (Philips, Best, The Netherlands). The authors use SPACE imaging, which can be obtained with and without fat suppression and can generate various contrasts depending upon the clinical situation: T1, proton density (PD), T2, SPAIR (spectral adiabatic inversion recovery), and STIR (short tau inversion recovery). The 3D SPAIR SPACE is used in extremities due to its higher SNR, 3D STIR SPACE is used for plexuses due to better and more homogeneous fat suppression, and 3D T2 SPACE is used for spine evaluation (also referred to as MR myelography) and for the evaluation of preganglionic nerve segments, as they exit from the spinal cord (Figure 1). Three-dimensional CISS (constructive interference in steady state) is another technique available for high resolution cranial nerve and pre-ganglionic segment evaluation in the background of high-intensity CSF [19]. While 3D imaging does not currently replace the 2D imaging (in plane resolution 0.4-0.5 mm) for smaller nerves, it is useful for multiplanar demonstration of pathology to the referring physicians for presurgical planning (Figure 2) [13]. The 3D SPAIR SPACE and 3D STIR SPACE approaches require postprocessing to remove the hyperintense vessels and fluid containing structures, which often contaminate the field of view and may lead to difficulties in nerve visualization (Figures 3(a) and 3(b)). 

## 4. Three-Dimensional Anatomic Nerve-Selective MR Neurography

**Figure 4:**
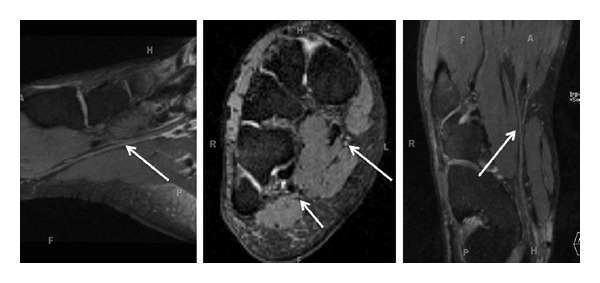
3D anatomic nerve selective MRN-Multiplanar reconstructions from an isotropic 3D DW PSIF sequence through the mid foot shows the normal long axis appearances of the medial plantar nerve (large arrows) and lateral plantar nerve (small arrow).

Nerve-selective MR imaging is aimed at suppressing the vascular and/or fat signal to create unique tissue-selective images. These are especially useful for radiologists and referring physicians who are not used to looking at peripheral nerve images on a routine basis. Such techniques should be incorporated in the MRN protocol whenever accurate nerve localization and/or presurgical assessment are required. There are various ways to create such images, with all techniques employing some sort of diffusion weighting. The peripheral nerves have a strong longitudinal order and orientation of structural components, leading to diffusion anisotropy. Thus, addition of diffusion weighting (DW) to the anatomic sequence (hybrid technique), or with predominant use of a directionally encoded DW sequences, the technologist can easily generate selective longitudinal images of the peripheral nerves due to the predominant and unrestricted longitudinal diffusivity of protons. One such technique optimized by the author (AC) and is routinely used at his institution is fat suppressed 3D DW PSIF (reversed fast imaging in steady state free precession) due to its ability to produce nerve specific images with excellent vascular suppression (inherent low diffusion moment, *b* value ~80 s/mm^2^) and fat suppression, while retaining all the advantages of an isotropic 3D MR imaging technique (Figure 3(c)). It should be noted that 3D DW PSIF is a sensitive sequence; it is prone to motion and ghosting artifacts. However, MIP reconstructions can enhance apparent SNR and be used to elegantly demonstrate nerves in multiple planes (Figure 4) [3, 6, 20]. Since it is a steady state sequence with both T1 and T2 contrasts, it shows neuropathy and mass lesions as abnormal T2 hyperintensity, with normal nerves appearing isointense to muscles, similar to other anatomic spin echo type sequences. Additionally, it can also be used before and after intravenous contrast and has potential to replace traditional fat suppressed postcontrast T1W imaging. 

**Figure 5:**
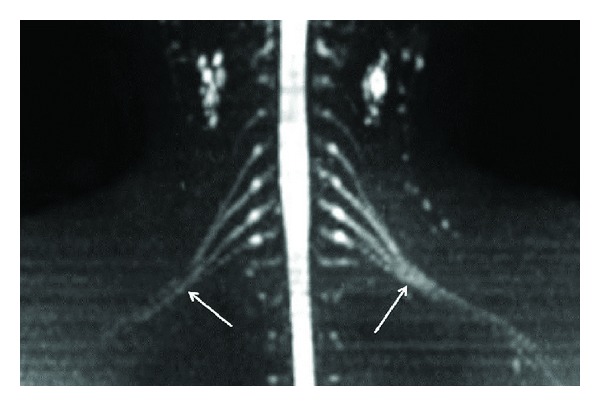
3D anatomic nerve selective MRN-Coronal MIP from a 3D diffusion weighted single shot EPI (*b* value 800 s/mm^2^) shows the symmetrical normal appearance of bilateral brachial plexuses (arrows).

**Figure 6:**
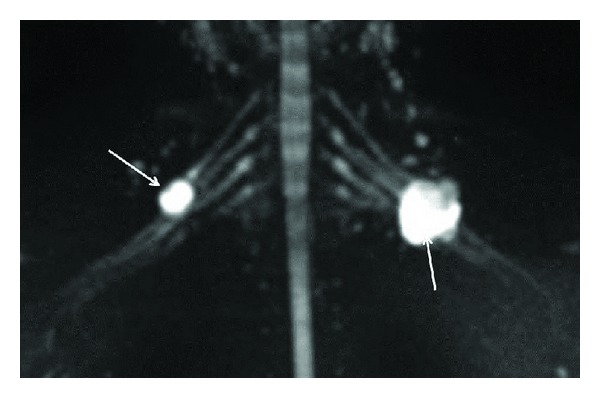
3D anatomic nerve selective MRN-Coronal MIP from a 3D diffusion weighted single shot EPI (*b* value 800 s/mm^2^) shows the peripheral nerve sheath tumors originating from the right C5 nerve root and the left upper trunk (arrows).

Selective DW peripheral nerve imaging requires a higher diffusion moment (*b* value ~400–1200 s/mm^2^). It can be performed with a unidirectional motion probing gradient (*b* value ~800 s/mm^2^) applied in anteroposterior direction in the axial plane using single shot STIR echo planar imaging. The perpendicular positioning of the motion probing gradient to the nerves offers the highest signal as the diffusion is relatively more restricted across the nerve (Figures 5 and 6) [21]. Another recently described technique known as SUSHI (subtraction of unidirectionally encoded images for suppression of heavily isotropic objects using an intermediate *b* value ~400–500 s/mm^2^) depicts the peripheral nerves with relative selectivity along their long axis by subtracting DW datasets acquired in directions parallel and perpendicular to the peripheral nerves using a pair of diffusion sensitizing gradients [22]. As opposed to diffusion tensor imaging (DTI), these images maintain relatively higher SNR due to a limited number of motion probing gradients (less than 3). The acquired data can be reconstructed using thick slab MIPs followed by grey scale window setting inversion to selectively depict the long trajectory of the nerves with decreased noise conspicuity. The MIPs are best performed using a soap bubble technique (Philips Healthcare) that uses a curved subvolume data set [21, 22], which helps exclude overlapping structures. Although this method is useful for selective nerve depiction, we prefer 3D anatomic and hybrid 3D DW PSIF images over DW imaging as these are better for fascicular evaluation and regional neuromuscular assessment due to the higher SNR and resolution available on these techniques. Additionally, pre-ganglionic nerve segment assessment as well as evaluation of the nerves running parallel to the motion probing gradient are difficult to perform on DW imaging. Finally, bones and lymph nodes show up as high signal on DW imaging and vessel contamination may still be present, leading to difficulty in nerve visualization, especially in the distal calf.

## 5. Three-Dimensional (3D) Functional Nerve Selective Imaging

The anatomic techniques are good for the assessment of neuropathy and worsening axonal degeneration; however, T2 signal alterations are not proven to reliably assess the nerve regeneration after traumatic injuries or nerve surgery [24–26]. The need for more objective and noninvasive functional measures of axonal regeneration in peripheral nerves is increasingly recognized as new therapeutic targets are being explored and many novel interventions are entering the preclinical stage of drug development. Three-dimensional (3D) functional nerve selective imaging can be performed using diffusion tensor imaging (DTI), and results from initial feasibility studies are becoming available [27–29].

**Figure 7:**
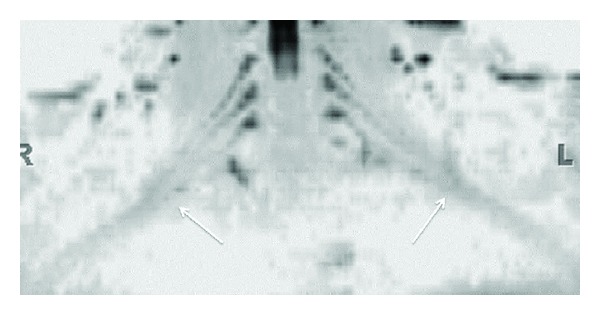
3D functional nerve selective MRN-Coronal MIP (inverted grey scale contrast) from a 3D DTI using single shot EPI (*b* value 0, 800, 1000 s/mm^2^ and 12 directions of interrogation) shows the symmetrical normal appearance of bilateral brachial plexuses (arrows). However, notice the decrease in SNR as compared to diffusion weighted imaging as shown in Figure 5.

**Figure 8:**
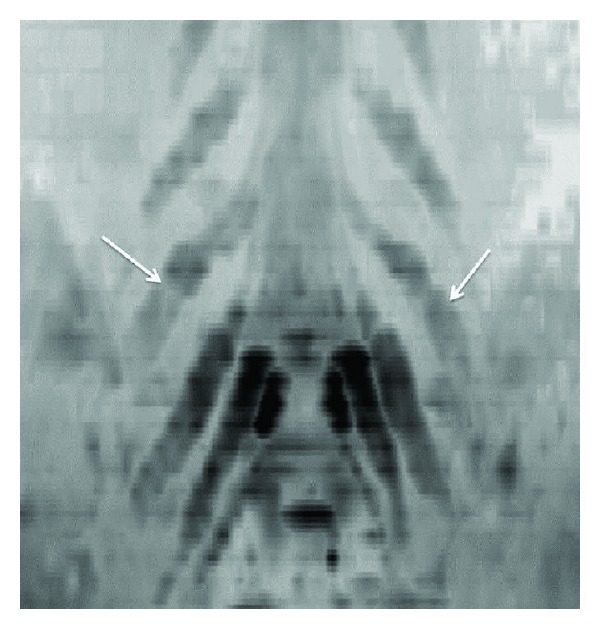
3D functional nerve selective MRN-Coronal MIP (inverted grey scale contrast) from a 3D DTI using single shot EPI (*b* values 0, 600 s/mm^2^ and 20 directions of interrogation) shows the symmetrical enlargement of bilateral LS plexus nerve roots (arrows) in a known case of hereditary motor and sensory neuropathy with diffusely reduced FA values in bilateral nerves.

DTI interrogates the random thermal motion of water molecules within tissues along different axes. It can therefore be used to exploit the potential of distinct microstructural properties of the peripheral nerves that hinder proton diffusion in some directions and facilitate it in other directions. DTI thus allows interrogation of the microarchitecture of the nerves, which is beyond the resolution of anatomic imaging techniques. This technique has proven clinical utility in a number of brain and spinal cord studies. However, early feasibility data from peripheral nerve are becoming available in the last few years with encouraging results in carpal tunnel studies in humans and sciatic nerve studies in animals [5, 27–29]. Generally, 6 or more diffusion encoding directions are required for DTI and commonly 12–20 directions and several *b* values (commonly 0, 500, 1000 s/mm^2^) are used to obtain improved diffusion tensor maps. However, this may result in significantly reduced SNR (Figure 7). Poor fat saturation, image distortions, as well as eddy current and ghosting artifacts may limit image quality. Additionally, acquisition times increase predisposing the imaging to motion artifacts. Three Tesla imaging with parallel acquisition, imaging in the axial plane, and tighter echo spacing enable highly resolved tensor maps. For peripheral nerves, higher *b* values in the range of 800–1000 s/mm^2^ and for plexuses, lower b values in the range of 600–800 s/mm^2^ generally produce adequate SNR and good quality datasets (Figure 8) [30]. However, the choice of the *b* value ultimately depends on the field strength, coil, and receiver technology.

DTI not only allows selective nerve visualization, but also enables quantitative measurement by creation of tractography maps. The measurements are fitted to a 3D diffusion ellipsoid tensor model [31, 32] which allows calculation of several semiquantitative parameters including the apparent diffusion coefficient (ADC) and fractional anisotropy (FA). ADC is a scalar value that reflects molecular diffusivity under motion restriction, and FA characterizes the directional variability in the diffusion, with an FA value of 0 representing random isotropic diffusion and 1 representing complete anisotropy. Fiber tractography can be performed with a line propagation technique (linear tracking algorithm) in which a tracking line is propagated from a start point (seed) placed perpendicular to the nerve in the principal diffusion direction. This method excludes the other false positive trajectories that may arise from anisotropic structures, such as the skeletal muscles. DTI can be used to evaluate fiber density by displaying tracts on color-coded 3D images [28]. 

Normal nerve FA values range from 0.4–0.8 depending on the SNR and type of nerve. However, the mean values of the normal nerves under interrogation are generally within 2 standard deviations of the adjacent nerves or contralateral equivalent nerves, and significant reductions of FA in abnormal nerves have been observed [27, 31]. Increased diffusivity, measured using the ADC, generally reflects inflammation, edema, or expanded nerve space, whereas decreased FA may be caused by damaged tissue microstructure, axonal loss and/or demyelination, or increase in isotropic water volume. Therefore, decreasing FA values is expected with worsening neuropathy. In a study by Takagi et al., the FA values decreased at the site of injury immediately following the insult and distally along the nerve 4 days after the injury. Additionally, increased FA values were observed at 3 weeks after injury correlating with functional motor and sensory recovery as well as nerve regeneration [7]. The previous authors found that the FA alterations of the peripheral nerves were more strongly correlated with axon-related (axon density and diameter) than with myelin-related (myelin density and thickness) parameters, supporting the theory that axonal membranes play a major role in anisotropic water diffusion and that myelination can modulate the degree of anisotropy. Tractography based on FA values also showed loss of fiber tracking distal to the crush site at earlier time points, and recovery of fiber tracking was noted at later time points related to axonal regeneration. In another sciatic nerve crush model, Lehmann et al. also found that FA values are a more useful parameter than diffusivity in the assessment of axonal regeneration [33]. In a recent carpal tunnel syndrome (CTS) case-control study, the FA, radial diffusivity, and ADC differed significantly between healthy subjects and CTS patients. DTI indices differed in regions proximal to and within the carpal tunnel in a manner which seemed to appropriately reflect pathophysiology of CTS [34].

**Figure 9:**
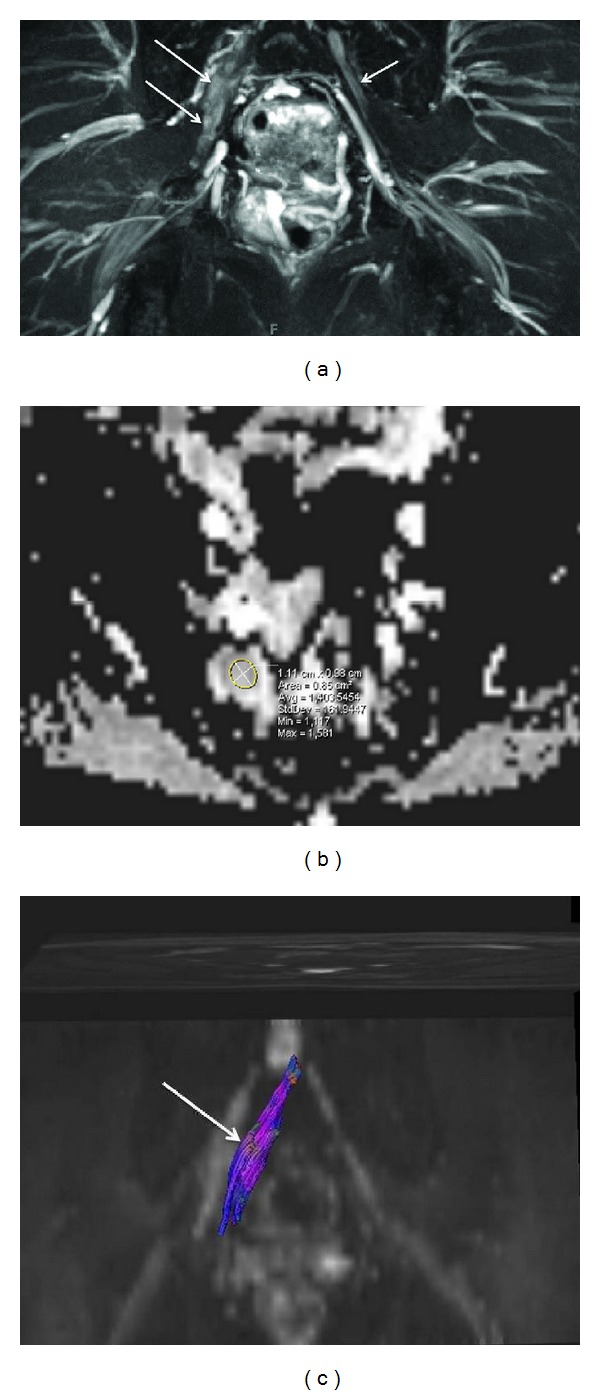
(a) Combined anatomic and functional MRN-MIP reconstruction from a 3D STIR SPACE sequence shows abnormal fusiform enlargement of the right S1 nerve root by a benign peripheral nerve sheath tumor (large arrows). Notice the normal appearance of the contralateral S1 nerve root (small arrow). (b) Combined anatomic and functional MRN-ADC map from single shot EPI DTI (*b* value 0, 600 s/mm^2^ and 20 directions) sequence shows high ADC value (1.4 × 10^−3^ mm^2^/s). (c) Combined anatomic and functional MRN-Tractography map following tensor calculation from single shot EPI DTI sequence shows nearly normal tracts (arrow) through the mass lesion in keeping with benign nature of the lesion.

**Figure 10:**
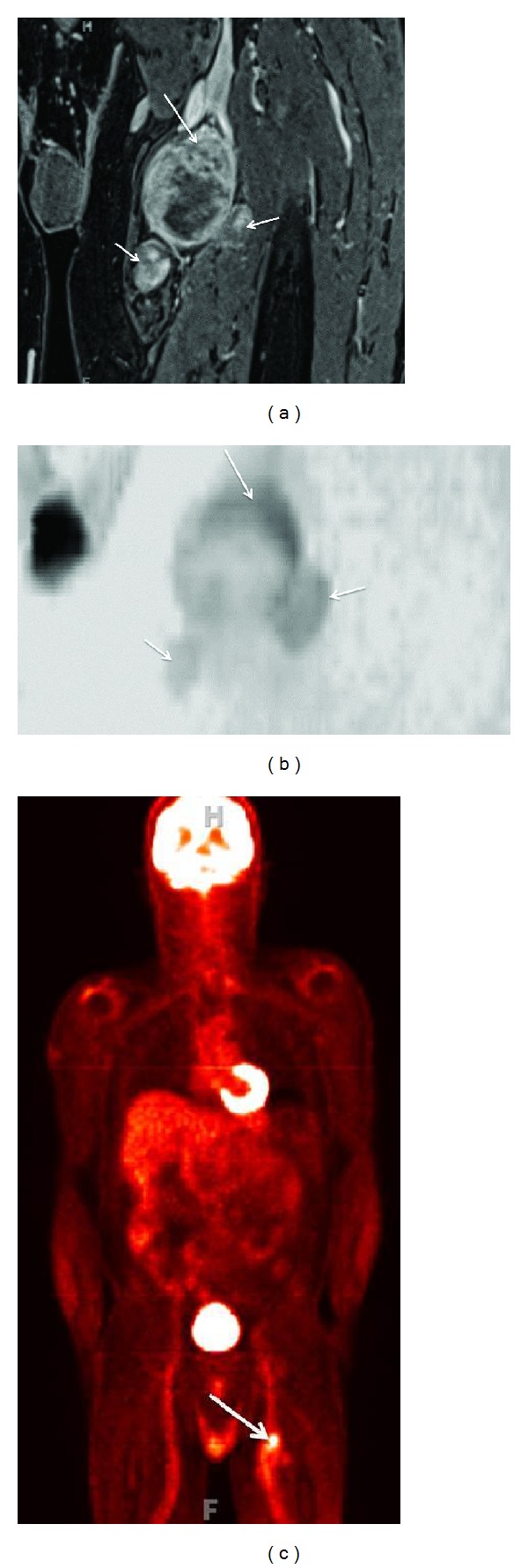
(a) Anatomic MRN, functional MRN, and F-18 FDG-PET correlation-Coronal fat suppressed postcontrast 3D T1W image of a biopsy proven malignant peripheral nerve sheath tumor shows the solid heterogeneous enhancement along the superior aspect of the lesion (large arrow). Notice additional smaller benign PNSTs in this case of neurofibromatosis type I (small arrows). (b) Anatomic MRN, functional MRN, and F-18 FDG-PET correlation-Coronal MIP reconstruction (inverted grey scale contrast) from single shot DTI (*b* values 0, 800, 1000 s/mm^2^, 12 directions) image shows the lowest ADC values (0.7 × 10^−3^ mm^2^/s) along the superior aspect of the lesion, which was targeted for the successful biopsy (large arrow). Notice that the additional smaller benign PNSTs show higher ADC values (small arrows). (c) Anatomic MRN, functional MRN, and F-18 FDG-PET correlation-Coronal F-18 FDG image shows high SUV max value (4 increased to 6 on delayed image) in the corresponding superior aspect of the lesion.

ADC values also help differentiate among benign and malignant tumors. Higher ADC values are seen with benign peripheral nerve sheath tumors and other soft tissue tumors (1.1–2.0 × 10^−3^ mm^2^/s), while low diffusivity values (0.7–1.0 × 10^−3^ mm^2^/s) are seen in malignant lesions (Figure 9) [35]. We presently use 3 cutoffs for their clinical practice currently (ADC—<1.0 × 10^−3^ mm^2^/s—suspicious for malignancy, ADC—1.0–1.5 × 10^−3^ mm^2^/s—indeterminate, ADC—>1.5 × 10^−3^ mm^2^/s—usually benign.) However, the ADC values should complement the conventional imaging appearances of these lesions, and one should not read them in isolation. Secondly, the reader should focus on the lowest ADC of the lesion, which correlates with the cellularity of the lesion, since even malignant lesions can have higher ADC values in their necrotic/less cellular portions. The lower ADC areas also correlate with the highest F18 FDG PET uptake regions and solid nodular arterial enhancing areas of the tumors, which helps in locating the most cellular areas for biopsy (Figure 10). It should also be kept in mind that interval measurements over time are more useful than a one-time measurement. This helps to follow the lesions, while patient is on medical treatment, such as imatinib. Increasing ADC values generally correlates with tumor response; however, it remains to be seen if they add more value over contrast imaging. But, as initial results are becoming available, MRN with DTI promises to be a single shot modality for the evaluation of peripheral nerve sheath tumors. Finally, tractography provides another insight into the pathophysiologic mechanisms of the lesions arising within or in close relationship to the directionally ordered nerve fibers. The degree of nerve tract disruption potentially correlates with increased aggressiveness/malignancy of the lesion and gives the surgeon an idea about potential fascicular infiltration of the tumor cells and difficulty in resection, which can be foreseen before the operation and is currently beyond the capability of anatomic images [1, 28].

DTI provides a unique opportunity to evaluate the complex diffusion patterns that occur within the nerve and allows understanding of pathophysiologic mechanisms in a 3D manner although some limitations need to be understood. DTI is a sensitive sequence, prone to motion and ghosting artifacts. Although peripheral nerves have a simpler architecture than the central nervous system and tractography should be easier to perform, DTI usually suffers from the relatively short T2 of water protons in the nerves leading to poor SNR. The variations in image quality or DTI parameters may significantly degrade serial assessment of parametric values and tensor maps. Maintaining the same acquisition parameters on serial scans, the application of good uniform fat suppression (water selective/Dixon/STIR), parallel imaging, keeping the slice thickness at or above 4 mm, employing higher strength gradients, lower echo times, and tighter echo spacing each help to generate acceptable images with adequate spatial resolution and reproducible tractography. The reader should understand that DTI is still at a feasibility assessment stage and is limited by relative lack of standardization and complexity of post-processing techniques. The preliminary ADC and FA data among different subjects show high (274%) variability. However, one could use healthy contralateral nerve as an internal control, since there is absence of statistically significant intrasubject side-to-side variability in quantitative data [29]. 

Fixed points of measurements based on bony landmarks should be used for quantitative measurements to mitigate variability, and the applied regions of interest should be small to avoid partial volume artifacts and large enough to cover the representative area of the nerve. Finally, the reader should understand that axonal loss injury also results in myelin loss over time, and the exact relative contributions of the two pathological conditions to the DTI parameters measured are not clearly known. With future research, DTI will likely play an important role in the evaluation and further understanding of neuromuscular diseases and is expected to be an important complement to current anatomic MRN protocols. 

## 6. Magnetization Transfer (MT) Imaging

MT imaging provides information about *in vivo *tissue integrity by exploiting differences in the properties of free water protons and restricted protons that are bound to macromolecules, proteins, and cell membranes. The bound protons in peripheral nerve include collagen, myelin, and membranes contained in the nerve fibers. The bound protons have a T2 relaxation time too short to be detected by conventional MR imaging techniques. However, MT imaging offers indirect characterization of the bound proton component and might provide information about nerve damage and demyelination in peripheral neuropathy. Selective saturation of the bound pool can be achieved with an applied off resonance radiofrequency pulse and when combined with a 3D spoiled gradient-echo image readout may be used to generate additional contrast on conventional MR images. The semiquantitative MT ratio (MTR) may also be calculated by comparing the signal reduction of an MT-weighted image relative to an image without MT saturation [36]. 

**Figure 11:**
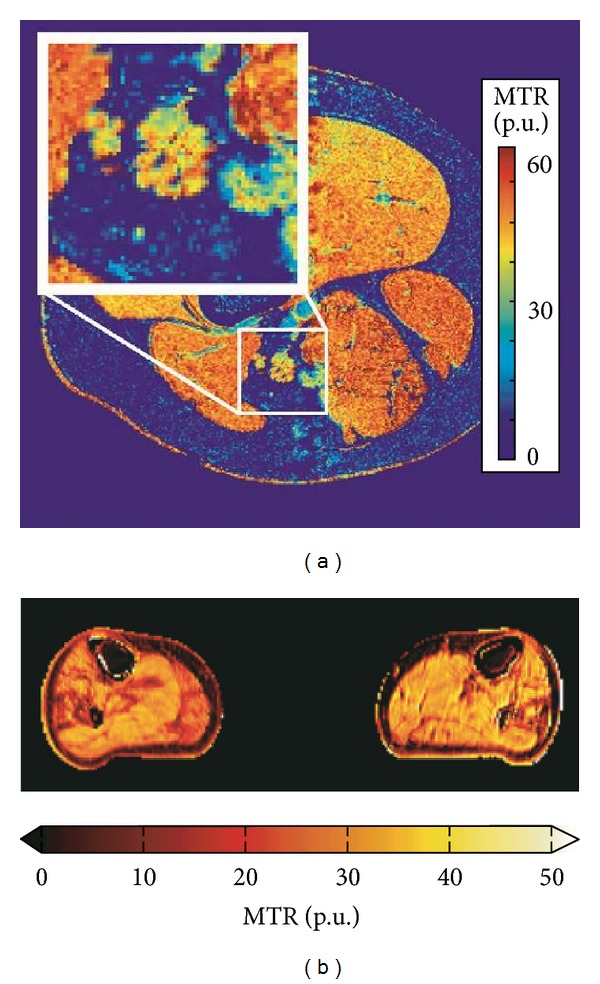
(a) MT neurography imaging of the sciatic nerve- Magnetization transfer ratio map at the mid-thigh level. Inset: enlargement of the sciatic nerve demonstrates measurable MTR effect in the nerve. (b) MT muscle imaging of neuropathy. Magnetization transfer ratio map of the mid-calf muscles of a patient with Charcot-Marie-Tooth disease type 1A (CMT1A). The MTR is reduced in areas affected by pathology. The maps have been corrected for RF inhomogeneities with a compensation method using B1 transmit maps [23].

In isotropic fluid compartments such as the cerebrospinal fluid (CSF), where a negligible concentration of macromolecules is present, the MTR is close to zero. In studies of multiple sclerosis, a decrease of MTR baseline values has been observed to correlate with demyelination, edema, and/or tissue damage in the brain [37]. In a study of optic neuropathy, MTR was reduced in affected nerves compared to clinically unaffected nerves [23]. Retinal neuroaxonal loss also correlated with MTR. Since axonal loss following optic neuritis also results in myelin loss, the relative contributions of the two pathological conditions to the MTR measures could not be evaluated. In a recent study of healthy volunteers, Gambarota et al. observed significant differences in the MTR of foot nerves versus median nerves; however, the regional muscles showed no significant differences [36]. MT imaging of muscle tissue has shown promise in neuropathic diseases including diabetic foot neuropathy, chronic inflammatory demyelinating polyneuropathy (CIDP), and Charcot Marie Tooth (CMT) disease (Figure 11), where decreased MTR is observed in muscles affected by these diseases as well as in muscle conditions including limb girdle muscular dystrophy [23, 38–41]. More research on MT imaging in peripheral neuropathy is needed, and its value for evaluation of muscle denervation compared with STIR and DW imaging needs to be proven before it can be used in the routine clinical setting. MT imaging has limitations that include a typically high sequence specific absorption rate (SAR) and a dependence of the measured MTR values on the specific sequence parameters used. Quantitative measurements in nerve present a particular challenge because of the requirement for high spatial resolution and SNR.

## 7. Muscle Imaging

MR can demonstrate regional muscle denervation changes in addition to the direct signs of nerve abnormality. These muscle findings evolve from edema like signal on fat suppressed T2W MR images in acute stages to fatty replacement and atrophy with chronicity [18, 42]. Although the prognostic factors affecting the outcome of nerve repair vary depending upon the degree of nerve injury and other clinical factors, such as age, patient's expectation, and surgeon's experience, fatty replacement and atrophy of the regional muscles is one of the important and measurable factors negatively influencing functional and anatomic outcomes [12, 43]. In addition to DTI and MT imaging mentioned earlier, a number of other functional MR imaging tools are available for assessment of regional muscle architecture and perfusion such as MR spectroscopy (MRS), C13 hyperpolarized imaging, or multiecho imaging for fat fraction assessment, chemical shift imaging (CSI), as well as ASL (arterial spin labeling) and BOLD (blood level oxygenation determination) imaging. Research is under way using these technologies at various centers looking at different functional and pathophysiologic mechanisms of the neuromuscular disease [44–48]. Despite the advantage of MR imaging in detecting muscle morphology changes, biochemistry, and pathology combined with the potential to influence the way surgeons approach and treat nerve injury patients, there is currently paucity of research or literature on this subject on how it would affect clinical decision making.

## 8. Whole Body MRN

**Figure 12:**
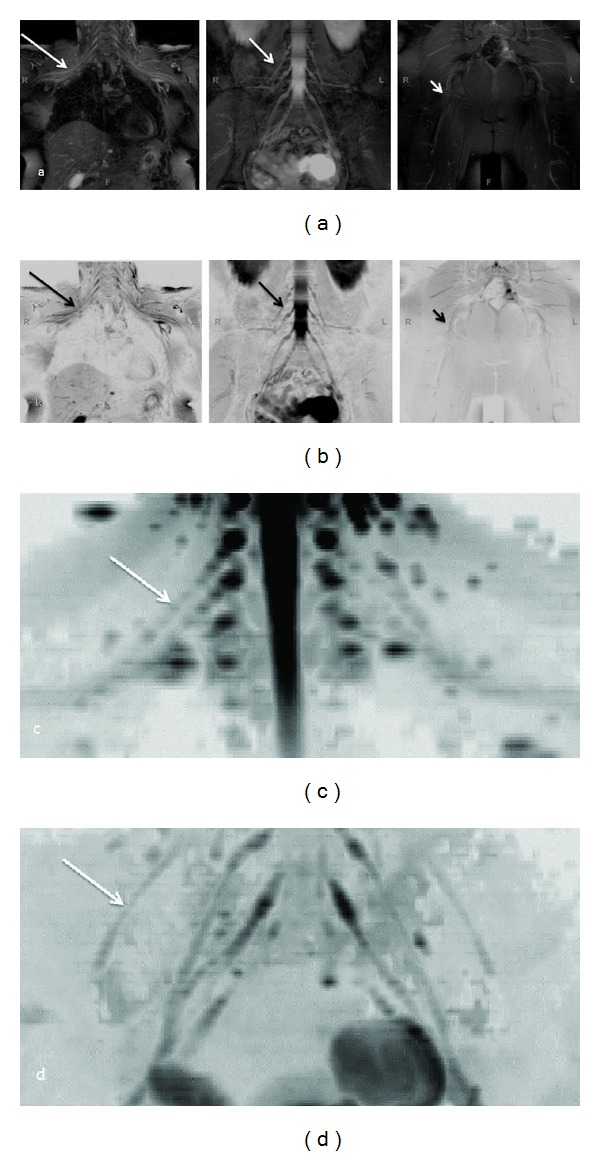
(a), (b) Whole body MRN: Coronal MIP reconstructions from 3D STIR SPACE sequence ((a) conventional window setting; (b) inverted window setting) show the normal appearance of bilateral brachial plexus (large arrows), LS plexus (medium arrows), and sciatic nerves (small arrows). (c), (d) Whole body MRN: Coronal MIP reconstructions (inverted grey scale contrast) from single shot DTI sequence (*b* values 0, 600 s/mm^2^, 20 directions) of the brachial plexus (c) and LS plexus (d).

Whole body MR imaging has been around for many years for the evaluation of metastatic disease, myeloma, and lymphoma. Takahara et al. described a free breathing DW whole body MR imaging technique with background body signal suppression and an STIR prepulse for robust fat suppression, which enables a longer effective imaging time than that with breath-hold imaging and allows acquisition of a large number of thin sections and three-dimensional analyses. This allowed signal decrease from intravoxel incoherent motion and not from respiratory motion (coherent motion) during the short time (approximately 100 ms) that motion-probing gradients are applied [49]. Whole body MR imaging using 3D isotropic STIR SPACE is also being performed with success at our institute [50]. Whole body MRN using simple diffusion weighted imaging was reported by Yamashita et al. in 2009 [51]. However, using a combination of thin collimation (1.5 mm isotropic) high contrast 3D STIR SPACE and DTI, whole body MRN can be performed in acceptable time periods of less than 45 min to 1 hour by employing parallel imaging technique (iPat factor: 2–4) with coverage from the skull base to the level of the knees in our preliminary experience, and feasibility studies are being performed using this technology (Figure 12). Adding DTI of the plexuses to the anatomic imaging represents a considerable advance in imaging and holds promise in assessment of disease burden and treatment response in neurocutaneous syndromes, CIDP, and hereditary neuropathies, such as CMT disease. This technique allows thin section reconstruction in any desired plane, avoids partial volume artifacts, and can easily detect thickened nerves or related mass lesions. Additionally, DTI of plexuses may be used for functional evaluation, and similar techniques could be used for followups on treatment or on finding worsening neuropathy.

## 9. Contrast Agents and Other Imaging Agents

The application of intravenous gadolinium-based contrast agents is currently limited in MRN studies to examinations performed for indications of infection, inflammation and neoplastic conditions (Figure 10) [2, 13, 20, 26]. Two different exogeneous intravenous contrast agents have been tried in evaluation of peripheral neuropathy. These include superparamagnetic iron oxide (SPIO) and gadoflouride M [52–54]. Gadoflouride M (GFM) is a nerve-selective contrast agent, which in animal studies has been shown to accumulate in nerve fibers undergoing Wallerian degeneration and disappear with remyelination. However, GFM has to undergo various research trials and human safety testing before it can receive FDA approval or can be used in clinical setting. Additionally, difficulty in visualizing axons makes these methods impractical for evaluating peripheral nerve injury in the present clinical scenario. Finally, molecular agents, such as stem cells, proteins, and nanoparticles under development can be potentially infused to selectively enhance/image nerve regeneration and hold considerable promise in the advancement of the field [14–16, 55].

## 10. Testing the Significance of Nerve Abnormality Causing Symptoms

**Figure 13:**
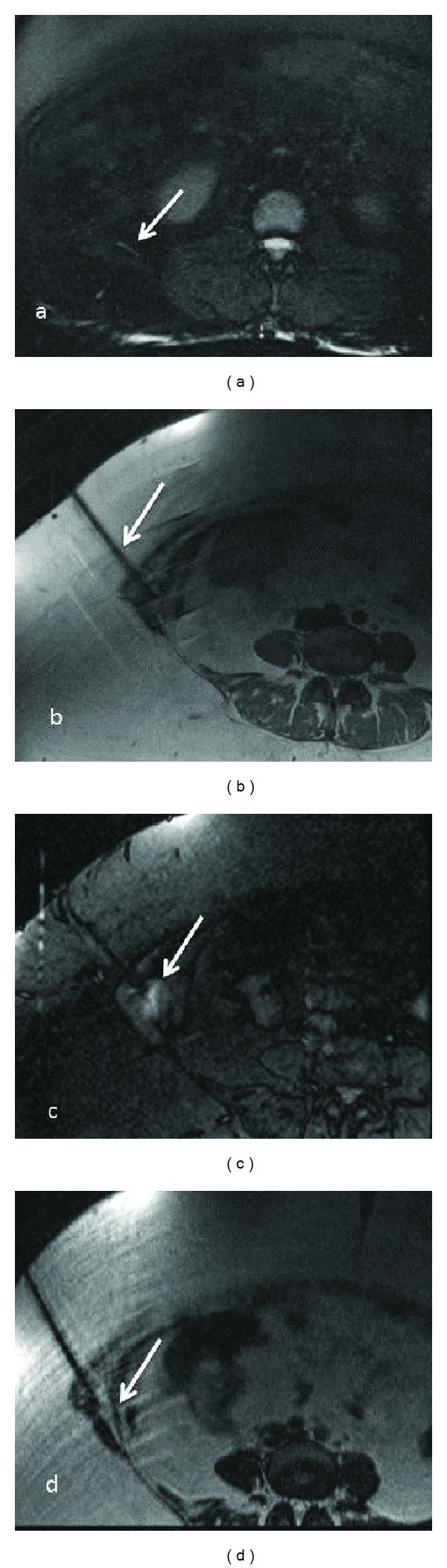
(a)–(d) MRN guided intervention-Axial T2 SPAIR (a), PDW (b), STIR (c), and T2 SPACE (d) images obtained during injection of local anesthetic and steroid combination around the right ilioinguinal nerve (arrow in a), beneath the external oblique aponeurosis. The patient experienced no significant pain relief following the injection in keeping with a negative block.

There are various ways to test whether the nerve itself or an associated imaging abnormality such as neuroma is the potential cause of patient symptoms. This could be done by ultrasound guided (USG) injections for superficial nerves and MR guided injection for the deeply located/nonidentifiable nerves on other imaging techniques (Figure 13). Although image guided injections have high face validity and are good for therapeutic effect, any diagnostic injection should ideally follow a protocol of comparative, graded, or placebo controlled injections to be accurate and valid [56, 57]. Another tool that may become available in future is high intensity focused ultrasound, which is shown to reliably induce sensations in human test subjects in a manner that correlates with their mechanoreceptor density [58]. 

## 11. Teaching and Web Tools

**Figure 14:**
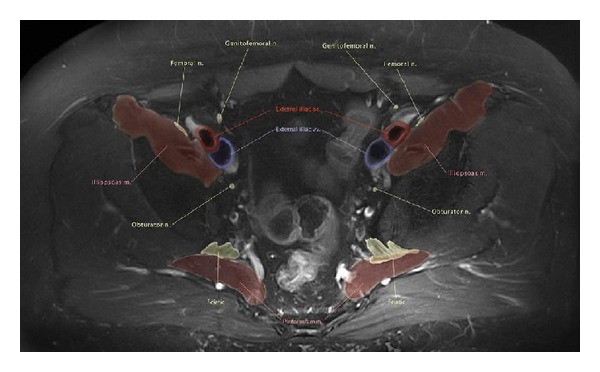
Anatomic illustration using MRN-Axial T2 SPAIR image through the upper pelvis with color drawing overlay nicely depicting the pelvic neuromuscular anatomy.

With excellent imaging of peripheral nerve anatomy and pathology available with current techniques and functional evaluation becoming possible, it is important for the radiologist to gain knowledge of the complex peripheral nervous system anatomy and its normal as well as abnormal imaging appearances. Also, with widespread use of high-resolution MRN imaging, various small and large nerve branches can be visualized. An experienced observer can easily find them; however, the complex anatomy can be difficult to navigate through for those not used to looking at these tiny peripheral branches. Existing illustrations are limited in their effectiveness due to previous technology, illustration technique, and limitations of education media. Increasingly, text books and journals are being replaced by digital interactive media. For this reason, it is important to superimpose anatomic illustrations over the MRN images and develop a digital platform for their demonstration to keep in stride with the advancements of science and education. With tandem depiction of sequential axial 2D MRN and 3D MRN images and original custom illustrated images with nerve labeling, the entirety of the peripheral nerve can be illustrated. Once integrated with the web tools, a reader can use them for their learning, correct nerve identification and MRN interpretation (Figure 14). Such an idea has been envisioned by the authors, and it is conceivable that it will likely become a useful web tool in few years to come.

## 12. Nerve Segmentation

**Figure 15:**
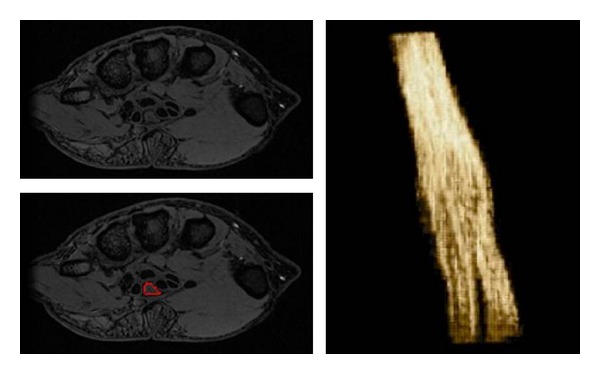
Nerve segmentation-Median nerve segmentation using custom made software. The tool allows semiautomated nerve segmentation, while a user draws region of interest on the nerve on sequential axial images.

Although appropriate performance of MRN using 3D imaging and the correct interpretation of the nerve findings is essential, it is also important to display the nerve anatomy and pathology to surgeons in a longitudinal manner, as they are used to seeing them during surgical dissection. Nerve segmentation allows longitudinal depiction of the peripheral nerves in multiple planes but also avoids contamination from the adjacent bright vessels and displays nerve architectural variations (bifid, trifid, or split). Most readers use axial images for primary detailed assessment of the nerves. A tool which can reconstruct the nerves in longitudinal planes by placing the regions of interest on the axial images, where these are best and reliably identified, would also help solve the issues encountered in the nerve identification, and depiction along its long axis and help diagnosis of displacement/entrapment/impingement by demonstrating alterations in the course as well as caliber of the nerves. Custom made software such as “Nerve Vision” will be available in future, which allows DICOM image import as well as navigation by implementing a semi-automatic segmentation algorithm consisting of two main steps. First, the user starts the segmentation of the object by interactively placing few control points at the nerve's border (not necessarily located on local maximal or most significant gradients). Second, these points are connected to an initial polygonal contour consisting of an order set of vertices. The algorithm then evolves a 3D polygonal contour that is as similar as possible to the landmarks in its behavior. In addition to the image-based attributes, the algorithm maintains the smoothness of the contour throughout the course of the nerve to ensure robust segmentation results. Additionally, in order to help the user best understand the volume data and to uncover important details in the volume data, the software implements a real-time visualization method based on GPU ray casting, which is capable of using multiple planes for convex volume clipping. This approach allows the user to select arbitrary number of clipping planes. This novel approach to 3D nerve segmentation of the peripheral nerves in the axial planes using semiautomated interactive algorithms provides realistic 3D visualization of the long axis of the nerve. This can be useful for identification, visualization of position, and for finding displacement/impingement and caliber variations of the nerve, as well as to track the nerve propagation through the confined spaces (tunnels) (Figure 15). 

To conclude, there have been considerable advances in the field of MR neurography making it a fruitful area for researchers to exploit the potential of a range of techniques currently available for imaging nerve and regional muscle and to assess their function in both research and clinical settings.
